# PERK inhibition prevents tau-mediated neurodegeneration in a mouse model of frontotemporal dementia

**DOI:** 10.1007/s00401-015-1487-z

**Published:** 2015-10-08

**Authors:** Helois Radford, Julie A. Moreno, Nicholas Verity, Mark Halliday, Giovanna R. Mallucci

**Affiliations:** MRC Toxicology Unit, Hodgkin Building, University of Leicester, Lancaster Road, Leicester, LE1 9HN UK; Department of Microbiology, Immunology and Pathology, Prion Research Center, Colorado State University, Fort Collins, CO 80523-1619 USA; Department of Clinical Neurosciences, University of Cambridge, Cambridge, CB2 0AH UK

**Keywords:** Unfolded Protein Response, Tau, Neurodegeneration, Dementia, PERK

## Abstract

The PERK-eIF2α branch of the Unfolded Protein Response (UPR) mediates the transient shutdown of translation in response to rising levels of misfolded proteins in the endoplasmic reticulum. PERK and eIF2α activation are increasingly recognised in postmortem analyses of patients with neurodegenerative disorders, including Alzheimer’s disease, the tauopathies and prion disorders. These are all characterised by the accumulation of misfolded disease-specific proteins in the brain in association with specific patterns of neuronal loss, but the role of UPR activation in their pathogenesis is unclear. In prion-diseased mice, overactivation of PERK-P/eIF2α-P signalling results in the sustained reduction in global protein synthesis, leading to synaptic failure, neuronal loss and clinical disease. Critically, restoring vital neuronal protein synthesis rates by inhibiting the PERK-eIF2α pathway, both genetically and pharmacologically, prevents prion neurodegeneration downstream of misfolded prion protein accumulation. Here we show that PERK-eIF2α-mediated translational failure is a key process leading to neuronal loss in a mouse model of frontotemporal dementia, where the misfolded protein is a form of mutant tau. rTg4510 mice, which overexpress the P301L tau mutation, show dysregulated PERK signalling and sustained repression of protein synthesis by 6 months of age, associated with onset of neurodegeneration. Treatment with the PERK inhibitor, GSK2606414, from this time point in mutant tau-expressing mice restores protein synthesis rates, protecting against further neuronal loss, reducing brain atrophy and abrogating the appearance of clinical signs. Further, we show that PERK-eIF2α activation also contributes to the pathological phosphorylation of tau in rTg4510 mice, and that levels of phospho-tau are lowered by PERK inhibitor treatment, providing a second mechanism of protection. The data support UPR-mediated translational failure as a generic pathogenic mechanism in protein-misfolding disorders, including tauopathies, that can be successfully targeted for prevention of neurodegeneration.

## Introduction

Endoplasmic reticulum (ER) stress and its associated Unfolded Protein Response (UPR) are emerging as major common themes in neurodegenerative disorders [13, 33]. The modulation of proteostasis is increasingly a potential therapeutic target in a wide range of human diseases associated with protein misfolding [12]. The UPR involves three major signalling cascades that enable the cell to deal with the build up of misfolded (or unfolded) proteins, which are detected by sensors within the ER [30]. One branch of the UPR, mediated by protein kinase RNA (PKR)-like/Pancreatic ER kinase (PERK), leads to the transient shutdown of protein synthesis. Activated (phosphorylated) PERK (PERK-P) phosphorylates the alpha subunit of eukaryotic initiation factor 2 (eIF2α); this inhibits the initiation of translation and induces expression of the transcription factor ATF4, and associated downstream signalling events, including induction of GADD34, the stress-induced eIF2α-P-specific phosphatase. (eIF2α can also be phosphorylated by other kinases as part of the integrated stress response (ISR), in response to cellular stresses such as viral infection and amino acid starvation). Increased PERK-P and eIF2α-P have been reported in postmortem analyses of brains from patients with Alzheimer’s disease (AD), Parkinson’s disease (PD) and the tauopathies Progressive Supranuclear Palsy (PSP) and Frontotemporal Dementia (FTD), as well as in prion disease [3, 15, 16, 37, 38]. In AD patients, UPR activation is thought to occur early in disease pathology in association with the accumulation of abnormal phosphorylated tau in neurons, but before neurofibrillary tangle formation [16, 26]. PERK-P staining has also been shown to be associated with phospho-tau-positive neurons in CA1 and dentate gyrus and subiculum regions of the hippocampus in FTD-tau cases [26].

However, it is unclear what the pathological significance of these findings is for the human diseases; specifically, whether they reflect activation of a protective mechanism or the signature of a pathogenic process (see Scheper and Hoozemans, for review [33]). This is of key significance for both mechanistic understanding and for development of potential treatments. Important insights come from animal models. PERK branch UPR overactivation occurs in several mouse models of neurodegenerative disease, including AD [1, 9, 13], prion [24], tauopathy [1, 18] and ALS [3, 32]. In prion-diseased mice, accumulation of misfolded prion protein (PrP) causes persistently high levels of PERK-P and eIF2α-P, and hence sustained translational failure. This results in a catastrophic decline in levels of key synaptic proteins, leading to synaptic failure and ultimately to neuronal loss [24]. This is true for both PrP overexpressing mice and for wild-type mice, supporting that PERK-eIF2α overactivation is not an artefact of high levels of protein expression, but occurs at endogenous levels (consistent with observations in humans). Critically, both genetic manipulations that reduce eIF2α-P levels [24] and pharmacological inhibition of PERK using the inhibitor GSK2606414 [4], or with the small molecule inhibitor, ISRIB, which acts downstream of eIF2α-P, restored vital protein synthesis rates and prevented neurodegeneration and clinical disease in prion-infected mice [11, 23, 24]. In contrast, pharmacological treatment preventing the reduction of eIF2α-P levels accelerated disease and exacerbated toxicity in prion-diseased mice [24]. Given the range of neurodegenerative diseases in which PERK branch UPR dysregulation is seen, it is crucial to understand its role in mediating neuronal loss in protein-misfolding disorders more broadly, particularly in AD and other dementias. This will also help determine its potential as a therapeutic target in these disorders. Wider relevance of the pathway in AD is supported by the fact that both PERK haploinsufficiency [9] and genetic suppression of other eIF2α kinases [21] rescue memory deficits and neurodegeneration in mouse models of AD. Indeed, neuronal eIF2α-P levels—and hence rates of protein synthesis—in wild-type mice are key to learning and memory [6], and pharmacological inhibition of eIF2α-P signalling boosts cognition [35]. Further, with relevance for AD and other tauopathies, PERK branch UPR activation is also known to contribute to the phosphorylation of tau—which is central to pathology in these diseases—both in vitro and in vivo [14, 20, 39]. In vitro, this has been shown to be due to PERK-mediated induction of glycogen synthase kinase-3 (GSK3β), a serine/threonine kinase that phosphorylates tau at disease-relevant epitopes [25], an effect that can be reversed by PERK inhibition [39]. Thus, UPR activation may have a dual pathological role in tauopathies, impacting on two processes fundamental to neurodegeneration in these disorders.

We have now examined the role of PERK pathway activation in the rTg4510 mouse model of FTD caused by the human tau P301L mutation [29, 31]. Mice positive for the mutant tau transgene (tau_P301L_^+^) show high levels of P301L tau expression throughout their lives, with age-related tau pathology, progressive memory deficits and, importantly, extensive forebrain neurodegeneration from 5.5 months of age [29, 31]. Tau_P301L_^+^ rTg4510 mice show abnormal conformations of tau from 2.5 months, with pre-tangle hyperphosphorylated tau detected by 4 months of age, with the appearance of mature neurofibrillary tangles and neurodegeneration in the hippocampus from 5.5 months. Neuronal loss becomes widespread throughout the forebrain after 7 months of age, leading to marked forebrain atrophy associated with clinical signs of poor grooming and motor impairment. Transgene-negative (tau_P301L_^−^) rTg4510 mice have normal brain morphology, behaviour and appearance and do not show phosphorylated tau [29, 31]. Increased levels of PERK-P in the brain of these mice have been reported during later disease stages (from 9 months), although the role of PERK-P signalling in mediating neurodegeneration in these animals has not previously been examined [1].

We first confirmed activation of PERK-eIF2α signalling in rTg4510 mice due to mutant tau expression. We then examined the effects of therapeutic inhibition of PERK signalling on both rates of neuronal protein synthesis and on tau phosphorylation and their role in neurodegeneration in these animals.

## Methods

### Mice

All animal work conformed to the UK Home Office regulations and institutional guidelines. rTg4510 tau_P301L_^+^ and tau_P301L_^−^ mice were obtained from Eli Lilly and Company. Treatment with doxycycline was performed from 4 months of age, by oral dosing as described [31]. rTg4510 mice were given two oral (bolus) doses of 10 mg/kg, then followed by Harlan Teklad T-7012, 200 diet with doxycycline 200 mg/kg in chow (*n* = 6 female mice). Treatment with GSK2606414 was by oral gavage twice daily with 50 mg/kg GSK2606414 suspended in vehicle (0.5 % HPMC + 0.1 % Tween-80 in H_2_O at pH 4.0) (*n* = 10 male), or with vehicle alone (*n* = 8 male) as described [23] from ~6 months of age.

### Western blotting

Protein extraction from hippocampi and immunoblots of UPR and synaptic proteins were performed as described [23, 24]. Proteins were detected with the following antibodies: eIF2α-P (S51) (1:1000; 9721, Cell Signaling) and eIF2α (L57A5) (1:1000; 2103, Cell Signaling), ATF4 (CREB-2, 1:1000; sc-200, Santa Cruz), GADD34 (1:1000, 10449-1-AP, Proteintech), PERK-P (1:200, 32577, Santa Cruz), PERK (1:1000, 3192, Cell Signaling), SNAP25 (1:10,000, ab5666, Abcam), PSD-95 (1:1000, EP2652Y, Millipore) total tau (Tau 5, 1:5000, AHB0041, Invitrogen), GSK3β (1:2000, 9832, Cell Signaling), pSer^9^-GSK3β (1:1000, 9322, Cell Signaling) and pTyr^279/216^-GSK3α/β (1:1000, 05-413, Millipore). Horseradish peroxidase (HRP)-conjugated secondary antibodies (1:10,000; DAKO) were applied, and protein was visualised using enhanced chemiluminescence (GE Healthcare) and quantitated using ImageJ. Antibodies against GAPDH (1:5000; sc-32233, Santa Cruz) and Beta-III-tubulin (1:5000, MAB1637, Millipore) were used to determine loading.

### Protein synthesis rates

Global translational levels (protein synthesis rates) were calculated by measuring ^35^S-methionine incorporation into proteins in acute hippocampal slices, as described [23, 24]. Briefly, slices were incubated with 5.7 mBq of ^35^S-methionine label in oxygenated artificial cerebrospinal fluid at 37 °C for 1 h. Incorporation of radiolabel was measured by scintillation counting (WinSpectral, Wallac Inc.). All biochemical analyses were performed in hippocampi from three to four mice.

### Histology

Paraffin-embedded brains were sectioned at 5 μm and stained with haematoxylin and eosin or NeuN antibody (1:200; MAB377, Millipore) for neuronal counts as described [23, 24]. CA1 pyramidal neuron counts were determined using three serial sections from three to four separate mice [24]. All neuronal counting was performed with the investigator being blind to the sample group being analysed. Immunohistochemistry for pSer^202^/Thr^205^ tau was performed using AT8 (1:100; MN1020, Thermo Scientific). Non-specific binding was blocked prior to primary antibodies using Histostain-Plus Bulk kit (Invitrogen). A biotinylated secondary antibody (Invitrogen) was used and stain visualised by diaminobenzidine reagent. All images were taken on using Axiovision 4.8 software (Zeiss) and counted using Volocity imaging system (Version 6.3) [24]. Immunofluorescence for pSer^202^/Thr^205^ tau was performed using AT8 (1:100; MN1020, Thermo Scientific) and PERK-P (1:50, 32577, Santa Cruz). Sections were blocked and permeabilized in blocking buffer (2 % donkey serum, 0.2 % Triton X-100 in TBS) for 1 h and then incubated overnight at 4 °C with primary antibody in TBS+2 % serum. Alexa Fluor 488 nm and 594 nm (1:500, Invitrogen) were then applied for 1 h. Slides were then mounted with VECTASHIELD Mounting media with DAPI (Vector laboratories). All images were taken using Zeiss LSM510 confocal microscope and counted using Volocity imaging system.

### Phospho-tau biochemical analysis

To detect levels of phosphorylated tau in total lysates AlphaScreen assays (Amplified Luminescent Proximity Homogeneous Assay Screen) were performed as described in [2] and as per manufacturer’s instructions. Phospho-tau-specific AT8 (pSer^202^/Thr^205^ tau) and PHF1 (pSer^369/404^ tau) antibodies were used. Briefly, optimised antibody/acceptor bead mix was incubated overnight at 4 °C with sample lysate or standard diluted in AlphaScreen assay buffer. Next, streptavidin-coated donor beads were added and incubated in the dark at room temperature for 4 h. Plates were read at excitation 680 nm and emission 520–620 nm using an Envision plate reader.

### XBP1-splicing assay

Total RNA was extracted from hippocampi with the mirVana isolation kit (Ambion). RNA samples were reversed-transcribed with oligo(dT) primers using ImProm-II Reverse Transcriptase (Promega). XBP1 mRNA was amplified with primers flanking the 26b intron (5′-GGAGTGGAGTAAGGCTGGTG and 5′-CCAGAATGCCCAAAAGGATA) using Phusin High-Fidelity taq polymerase (New England Biolabs) [23]. PCR products were resolved on 3 % agarose gels. MEF cells were treated with 5 μg/ml tunicamycin for 6 h and used as a positive control for XBP1 splicing.

### Statistics

All statistical analyses were performed using Prism V6 software using Student’s *t* test for data sets with normal distribution and a single intervention. ANOVA testing was performed using one-way analysis with Tukey’s post hoc test for multiple comparisons.

## Results and discussion

### High levels of total mutant tau expression lead to activation of the PERK branch of the UPR and sustained translational repression in rTg4510 FTD mice

**Fig. 1:**
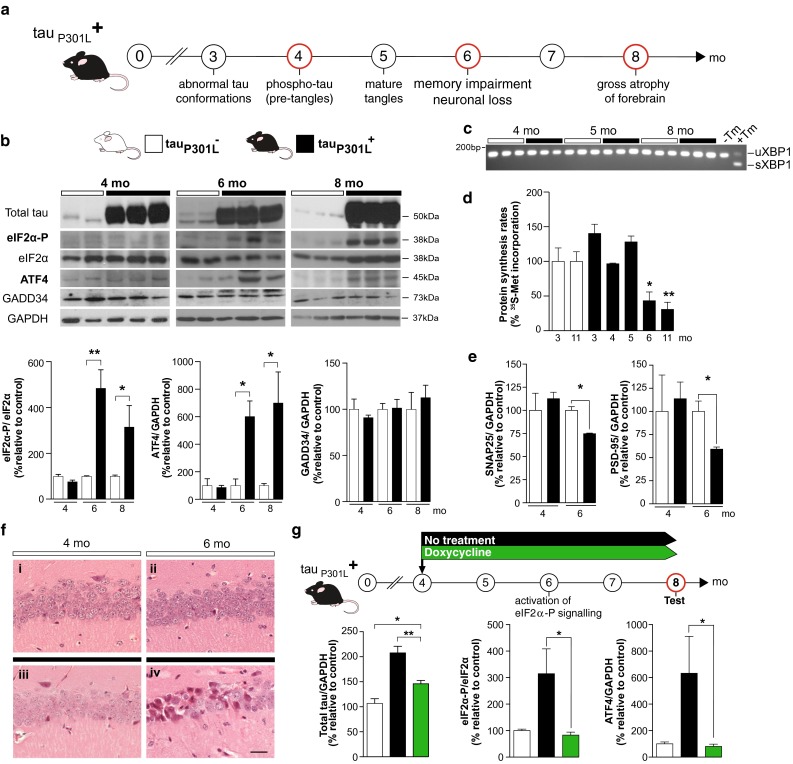
Mutant tau-expressing rTg4510 mice show overactivation of the PERK/eIF2α-P branch of the UPR resulting in a decline in protein synthesis rates by 6 months of age. **a** Scheme depicting disease progression in rTg4510 tau_P301L_^**+**^ mice from 3 to 8 months (mo). *Red circles* indicate times of testing. **b** tau_P301L_^+^ mice (*black bars*) show sustained elevated levels of eIF2α-P and ATF4 by 6 months of age, compared to levels seen in tau_P301L_^−^ (*white bars*). These remained elevated at 8 months, eIF2α-P and ATF4 were equivalent in both mutant tau-expressing and transgene-negative mice examined at 4 months. Levels of GADD34 do not change throughout disease progression and remain equivalent to tau_P301L_^−^ mice. Representative immunoblots of hippocampal lysates and bar charts quantitating relative levels of protein (*n* = 3) are shown. **c** RT-PCR of XBP1 transcript shows that there is no splicing in mutant tau-expressing and transgene-negative mice. Control lanes from untreated MEF cells (−Tm), or MEF cells treated with tunicamycin (+Tm) as a positive control for activation of IRE1 **d** Protein synthesis rates in hippocampal slices, determined by incorporation of ^35^S-methionine into protein, declined by ~57 % by 6 months of age in tau_P301L_^+^ mice in contrast to transgene-negative mice, consistent with eIF2α-P signalling (*n* = 3–4 mice per time point). **e** Levels of pre-synaptic protein (SNAP25) and post-synaptic protein (PSD-95) declined in parallel (*n* = 3, measured relative to GAPDH). **f** tau_P301L_^**+**^ mice showed neuronal loss in the CA1 region of the hippocampus by 6 months (panel iv) compared to age-matched tau_P301L_^**−**^ mice (*panel ii*). Representative images or hematoxylin and eosin-stained sections, scale bar 50 μm. **g** Doxycycline treatment from 4 months of age reduced total tau protein levels (*green bars*) compared to non-treated tau_P301L_^+^ mice (*black bars*) at 8 months of age, with accompanying reduction in eIF2α-P and ATF4 levels in 8-month-old tau_P301L_^+^ mice to levels similar to those seen in transgene-negative mice; and in contrast to high levels seen in untreated tau_P301L_^+^. Bar charts quantitating levels of total tau, eIF2α-P and ATF4 measured relative to GAPDH (in 3–4 independent samples) are shown. All bar charts show mean ± SEM, *p < 0.05, **p < 0.005, Student’s *t* test was used except in (**c**), where one-way ANOVA analysis with Tukey’s post hoc test for multiple comparisons was performed. *X*-axis represents increasing age in months.

We assessed PERK branch UPR activation in the brains of rTg4510 mice by measuring levels of eIF2α-P, ATF4, GADD34 and global protein synthesis rates over the course of disease. P301L transgene-expressing mice (tau_P301L_^+^) express high levels of mutant tau from birth (repressible by doxycycline administration [31]). Characteristically, they show memory impairment and hippocampal neuronal loss by 6 months, with widespread forebrain atrophy and overt clinical signs by 8 months [29, 31] (Fig. 1a). We tested mice from 4 months of age. All tau_P301L_^+^ mice expressed high levels of total tau compared to low levels in non-transgenic controls, which express only wild-type (murine) tau (Fig. 1b).

Levels of eIF2α-P and ATF4 were low in all animals until 6 months of age, by which time there was marked elevation of eIF2α-P and ATF4 levels in tau_P301L_^+^ mice compared to non-transgenic littermates (Fig. 1b), consistent with activation of PERK signalling. Interestingly, GADD34 levels did not increase over time (Fig. 1b), which is unexpected given induction of ATF4, but consistent with the effects of PERK branch overactivation seen in prion-infected mice [24]. The failure to induce GADD34 would explain the persistent high levels of eIF2α-P seen in tau_P301L_^+^ older mice (Fig. 1b), again similar to the sustained elevation of eIF2α-P levels seen in prion-diseased mice, both wild type and overexpressing [24]. We found no change in the other two signalling cascades of the UPR involving IRE-1-mediated XBP1 splicing (Fig. 1c) and cleavage of ATF6 (data not shown) throughout the time course of disease in rTg4510 transgene-positive mice, consistent with findings in prion-infected mice [23].

Consistent with PERK branch eIF2α-P activation, rates of global protein synthesis measured in brain slices of tau_P301L_^+^ mice had declined to ~40 % of those seen in non-transgenic littermates by 6 months of age, remaining repressed until 11 months (Fig. 1d), after which time the animals were killed. Protein synthesis rates were normal up to 5 months of age, suggesting the onset of sustained translational repression is between 5 and 6 months (Fig. 1d). Levels of the pre- and post-synaptic proteins SNAP25 and PSD-95 were equivalent in transgene-positive and -negative mice at 4 months, but reduced in tau_P301L_^+^ mice at 6 months (Fig. 1e), reflecting the drop in protein synthesis due to high levels of eIF2α-P at this time. As in prion-diseased mice [24], the onset of eIF2α-P-mediated translational repression was closely associated with onset of neurodegeneration at 6 months in rTg4510 mice, which is absent at 4 months (Fig. 1f).

To confirm that activation of PERK-eIF2α-P signalling is indeed the result of mutant tau expression in rTg4510 tau_P301L_^+^ mice, we tested the effect of repressing tau levels on eIF2α-P and ATF4 levels using doxycycline administration. Treatment of transgene-positive mice with doxycycline from 4 months resulted in marked reduction in total tau levels by 8 months (although not as low as levels in transgene-negative controls that express only murine tau) (Fig. 1g). This produced a corresponding marked reduction in eIF2α-P and ATF4 levels compared to levels seen in untreated tau_P301L_^+^ mice, and equivalent to those seen in non-transgenic mice (Fig. 1g). Our data are, therefore, consistent with UPR activation being a direct result of elevated levels of mutant total tau in these mice. Consistent with this, reduced PERK-P immunostaining after doxycycline treatment in rTg4510 mice has also been reported by others [1].

Thus, the onset of PERK/eIF2α-mediated translational repression is closely associated with synaptic protein loss and onset of neurodegeneration in mutant tau-expressing rTg4510 mice, similar to the temporal relationship between these events in prion-diseased animals [24]. Importantly, although it is true that levels of mutant tau overexpression are very high in this model, the pR5 mouse model of tauopathy in which levels of mutant tau expression are much lower, at ~70 % endogenous tau, also shows UPR activation [17, 18]. This is similar to the detection of PERK/eIF2α activation in prion-diseased mice, which is seen in both PrP overexpressing and wild-type mice [24], and is, therefore, unlikely to reflect an artefact of protein overexpression.

### PERK inhibitor treatment reduces eIF2α-P levels and restores global translation rates

Given the known neuroprotective effects of restoring protein synthesis rates in prion-diseased mice downstream of misfolded PrP accumulation [11, 23], we first asked if restoring translation through PERK inhibition in tau transgene-expressing mice was similarly neuroprotective. We treated tau_P301L_^+^ mice with the specific PERK inhibitor GSK2606414 [4] or vehicle alone from ~6 months of age, when eIF2α-P levels are known to be elevated (Fig. 1b) and neuronal loss is beginning (Fig. 1f; [31]). (These were proof-of-principle studies, as GSK2606414 is known to produce pancreatic toxicity despite profound neuroprotection after prolonged treatment in prion-diseased mice [23]. The question was whether similar neuroprotective effects of reversing translational repression—achieved through PERK inhibition—are seen in this model; not whether GSK2606414 is a viable treatment).

**Fig. 2:**
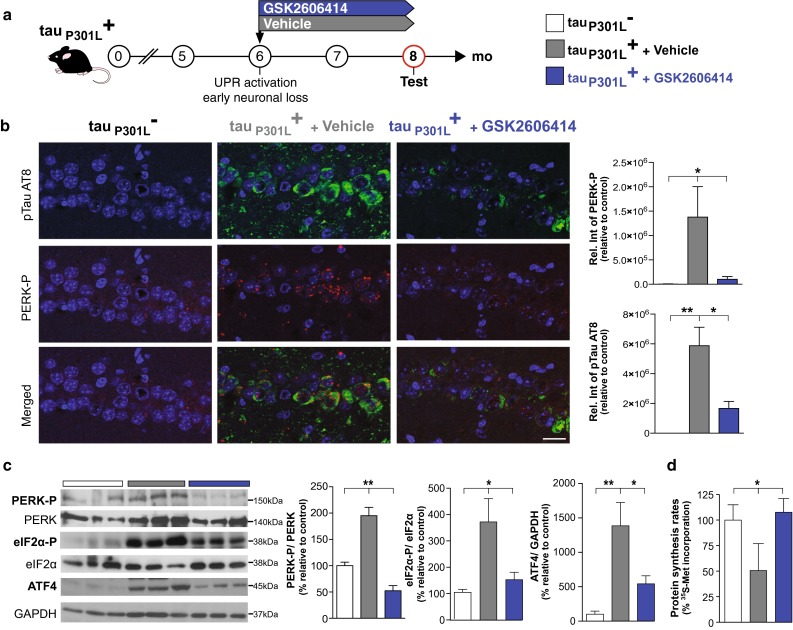
PERK inhibitor treatment reduces eIF2α-P and ATF4 protein levels and restores protein synthesis rates in mutant tau-expressing rTg4510 mice. **a** tau_P301L_^+^ mice were treated twice daily by oral gavage from 6 months with either the PERK inhibitor, GSK2606414 50 mg/kg, (*blue bars*) or vehicle (*grey bars*) and tested at 8 months of age. **b** Immunostaining showed a significant reduction in PERK-P (*red*) and pSer^202^/Thr^205^-tau (AT8, *green*) staining in the hippocampus after GSK2606414 treatment. Graphs show quantification of relative intensity for PERK-P and ptau compared to transgene-negative mice (*n* = 3–5 mice, scale bar 20 μm). **c** PERK inhibitor treatment markedly reduced PERK-P, eIF2α-P and ATF4 protein levels in 8-month-old tau_P301L_^+^ mice, preventing the decline of global protein synthesis rates as determined by ^35^S-methionine incorporation into protein (**d**) in comparison to vehicle-treated animals (*n* = 3 mice). Representative immunoblots of hippocampal lysates and bar charts quantitating protein levels (in three independent samples). All bar charts show mean ± SEM, *p < 0.05, **p < 0.01, using Student’s *t* test.

rTg4510 tau_P301L_^+^ mice received 50 mg/kg GSK2606414 (*n* = 10) or vehicle (*n* = 8) by oral gavage twice daily, a dose optimised for good levels of brain penetration, as described in [23], for 2 months (Fig. 2a). At this time point, at 8 months of age, animals were killed for analysis. As predicted, and consistent with previous observations in prion-diseased mice [23], GSK2606414 treatment significantly reduced levels of PERK-P, eIF2α-P and ATF4 levels in tau_P301L_^+^ mice, compared to vehicle-treated animals, which showed persistently elevated of levels of these proteins (Fig. 2b, c). Critically, PERK inhibitor treatment restored global protein synthesis rates to normal in 8-month-old tau_P301L_^+^ mice, in contrast to vehicle-treated animals, which showed markedly reduced translation rates at this time point (Fig. 2d). (GSK2606414 does not affect eIF2α-P levels and global protein synthesis where the UPR is not activated [23]).

### PERK inhibitor treatment is neuroprotective in FTD mice: reducing tau phosphorylation, and brain atrophy and abrogating clinical signs

**Fig. 3:**
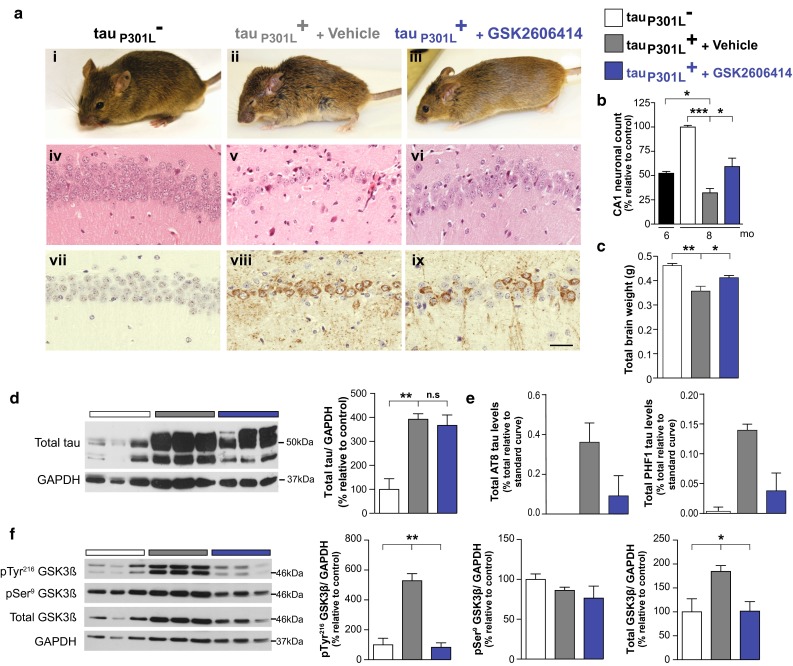
PERK inhibitor treatment decreases tau phosphorylation and prevents neurodegeneration and clinical disease in mutant tau-expressing rTg4510 mice. **a** GSK2606414 treatment prevented clinical signs in 8-month-old tau_P301L_^+^ mice, which showed normal grooming, posture and movement compared to vehicle-treated animals and were indistinguishable from transgene-negative animals of the same age (representative images, i–iii). Histologically, PERK inhibitor resulted in marked neuroprotection with preservation of hippocampal volume and CA1-3 neuronal ribbon (iv–vi, hematoxylin and eosin-stained sections), and immunostaining using AT8 showed a significant reduction in pSer^202^/Thr^205^-tau staining in the hippocampus after GSK2606414 treatment (vii–ix, representative images of hippocampal sections, scale bar 50 μm). **b** Average count of the number of CA1 pyramidal neurons in five consecutive slices from tau_P301L_^+^ treated with vehicle (*grey bars*) or GSK2606414 (*blue bars*) mice relative to control mice (tau_P301L_^−^, *white bars*) shows reduced loss of pyramidal neurons in PERK inhibitor-treated mice (i.e. prevention of neurodegeneration) to numbers similar to 6-month-old mutant tau-expressing mice (*black bar*) (*n* = 3–4 mice) (**c**) GSK2606414 partially prevented brain atrophy in comparison to vehicle-treated mice (*n* = 3–8 mice). **d** Total tau levels were not significantly different in PERK inhibitor or vehicle-treated animals (*n* = 3 mice). **e** AlphaScreen analysis with AT8 and PHF-1 shows a reduction in soluble phospho-tau at pSer^202^/Thr^205^ and pSer^396/404^ epitopes after PERK inhibitor treatment compared to vehicle-treated animals (*n* = 4 mice). **f** Levels of the active form, pTyr^216^-GSK3β, and total GSK3β levels increased compared to control mice. PERK inhibitor treatment significantly reduced pTyr^216^-GSK3β and total GSK3β levels in tau_P301L_^+^ mice to levels seen in control mice. Representative immunoblots of hippocampal lysates and bar charts quantitating protein levels (in three independent samples). All bar charts show mean ± SEM, *p < 0.05, **p < 0.01 ***p < 0.005, *n*.*s* non-significant, using Student’s *t* test.

Importantly, PERK inhibitor treatment was neuroprotective in rTg4510 mice, as in prion-diseased mice [23], partially restoring neuronal numbers, maintaining total brain weight and preventing clinical signs in 8-month-old animals, and preventing any progression of neuronal loss from 6 months of age, when treatment was begun (Fig. 3a–c). Thus, all vehicle-treated tau_P301L_^+^ mice (8/8) showed poor grooming, hunched posture, hind-leg clasping and/or poor mobility by 8 months of age (Fig. 3a, panel ii). In contrast, all PERK inhibitor-treated tau_P301L_^+^ mice (10/10) showed normal grooming, posture and movement at this stage (Fig. 3a, panel iii). Indeed, PERK inhibitor-treated tau_P301L_^+^ mice were indistinguishable clinically from their transgene-negative tau_P301L_^−^ littermates (Fig. 3a, panel i). Histological examination confirmed marked neuroprotection in tau_P301L_^+^ mice treated with GSK2606414, compared to vehicle-treated mice, which showed profound hippocampal neuronal loss (Fig. 3a, compare panels v and vi), characteristic of the extensive forebrain neurodegeneration described in these mice at this stage [31]. GSK2606414 treatment resulted in preservation of ~60 % of CA1 neurons at 8 months, compared to ~25 % in vehicle-treated mice at this time (Fig. 3b). The protective effect is notable especially given that treatment begun at 6 months, when neuronal loss is already beginning in the hippocampus (Fig. 1f; [29, 31]). On a macroscopic scale, PERK inhibitor treatment significantly reduced brain atrophy, with greater total brain weights compared to untreated transgene-expressing animals at this stage (Fig. 3c). Both the number of CA1 neurons and brain weights of PERK inhibitor-treated animals at 8 months were very similar to neuronal numbers reported in untreated tau_P301L_^+^ rTg4510 mice at 5.5 months by other workers [29, 31], supporting the fact that the compound prevented further progression of neurodegeneration. (All tau_P301L_^+^ mice treated with GSK2606414 developed signs of pancreatic toxicity after 2 months of treatment, as expected [11, 24], with weight loss and mild elevation of blood glucose levels).

We also examined the effect of PERK inhibition on pathological phosphorylated tau expression histologically and biochemically, which is central to pathology in all tauopathies [5]. We found that GSK2606414 treatment had no effect on soluble total tau levels at 8 months, which were indistinguishable from those of vehicle-treated mice (Fig. 3d). (This is not unexpected, despite the effect of PERK inhibition on global protein synthesis, because the P301L tau transgene is expressed under control of the PrP promoter [31], which has upstream open reading frames in the 5ʹ UTR that allow the protein to escape translational repression [24]).

However, levels of phospho-tau in rTg4510 transgenic mice brain were reduced by PERK inhibitor treatment. Tau can be phosphorylated at more then 30 different sites and hyperphosphorylation is crucial for aggregation and tangle formation. A number of different antibodies have been described that recognise different tau phosphorylation sites and pathology, including AT8 [10, 22], which detects phosphorylation at Ser^202^/Thr^205^ and is a marker for pretangles and tangles, and PHF1 [28], which detects late-stage tangles including tau phosphorylated at Ser^396/404^. Immunohistochemistry using AT8 showed a notable decrease in total phospho-Ser^202^/Thr^205^ tau staining in the CA1 region of the hippocampus in tau_P301L_^+^ mice after GSK2606414 treatment, with many neurons negative for AT8 staining and hence phosphorylated tau. In contrast, vehicle-treated tau_P301L_^+^ mice, showed prominent AT8-positive staining in the cell somata of almost all remaining CA1 neurons (Figs. 2b, 3a, compare panels viii and ix), consistent with the findings of Spires and co-workers showing prominent staining of PHF1 positive neurons in CA1 region of tau_P301L_^+^rTg4510 mice at 8.5 months of age [36]. Transgene-negative mice did not show any staining for phospho-Ser^202^/Thr^205^ tau (Figs. 2b, 3a, panel vii). Biochemical assays to quantitate reduction in total phospho-tau species in total lysates confirmed reduction in both pSer^202^/Thr^205^ (AT8) and pSer^369/404^ (PHF1) isoforms after PERK inhibitor treatment (Fig. 3e).

As discussed, the pathogenic role of UPR activation in tauopathies likely acts through two separate mechanisms. Thus, not only is PERK-mediated translational failure activated by high levels of misfolded/mutant tau, but UPR activation is known to also drive tau phosphorylation in vitro [14, 39] and in vivo [20], which can be prevented in vitro by PERK inhibition with GSK2606414 [39]. UPR-mediated tau phosphorylation is thought to occur, at least in part, indirectly, through the induction of the active form, pTyr^216^-isoform, of the serine/threonine kinase GSK3β that phosphorylates tau at disease-relevant epitopes [25]. In vivo, UPR activation activates GSK3β leading to tau phosphorylation in rat brains [20]; specific GSK3β inhibitors reduce tau phosphorylation and aggregation in mouse models of tauopathy [27, 34]. Further, in AD brains, the active form of GSK3β co-localises with phospho-tau [19] and with PERK-P [16], which is also seen in pR5 tau-expressing mice [17, 18]. We, therefore, assessed levels of active and inactive (pSer^9^-) forms of GSK3β, as well as total levels of the enzyme in tau_P301L_^+^ rTg4510 mice after PERK inhibitor or vehicle treatment. Consistent with other models, levels of active pTyr^216^-GSK3β, and total levels were increased in vehicle-treated tau_P301L_^+^ mice at 8 months, but these were reduced to levels seen in transgene-negative mice by PERK inhibitor treatment (Fig. 3f). The data are consistent with PERK inhibitor-mediated suppression of GSK3β activity resulting in reduced phosphorylation of tau and markedly lower levels pathological tau staining in the brains of treated animals compared to vehicle-treated controls. We noted that total levels of GSK3β increased in mutant tau-expressing mice (Fig. 3f). The reasons for this are unclear. *GSK3β* mRNA does not contain upstream open reading frames (uORFs) in its 5ʹ untranslated region (as does ATF4, for example), nor does it have relevant transcriptional regulatory sequences (data not shown).

Therefore, activation of the UPR may also result in persistent activation of GSK3β and contribute further to the neurodegenerative process by resulting in a vicious cycle of sustained UPR activation/tau phosphorylation. Treatments modifying PERK/eIF2α signalling may, therefore, be beneficial not only by restoring vital protein synthesis rates in compromised neurons, but also by decreasing the phosphorylation of tau, doubly reducing the toxic burden in these diseases. The data are of interest also because of opposing mechanisms in mouse models of specific familial ALS mutations and the rare peripheral neuropathy CMT1B, where genetic and pharmacological interventions that maintain high levels of eIF2α-P, rather than reducing it (at least in early disease) are protective [7, 8, 32, 40]. Thus, defining the effects of PERK pathway activation and its inhibition in a wide range of neurodegenerative disorders is crucial for effective intervention.

In conclusion, we have shown that UPR-mediated translational repression is associated with neurodegeneration in a mouse model of Frontotemporal Dementia, and that UPR activation also drives pathogenic tau phosphorylation in these mice. We have shown that neurodegeneration is significantly reduced that levels of phospho-tau decline and clinical disease is prevented by oral treatment with the PERK inhibitor, GSK2606414. The data support the conclusion that UPR dysregulation, at least in part, mediates tau neurodegeneration, as well as prion neurodegeneration [23, 24]. Given the prevalence of UPR activation in AD and related dementias, our data provide an increasingly compelling argument for the development neuroprotective therapies aimed at restoring neuronal protein synthesis rates, at least in part [11], and reducing phosphorylation of tau, for treatment of these diseases.
